# Tuberculosis: New Aspects of an Old Disease

**DOI:** 10.1155/2011/403623

**Published:** 2011-06-21

**Authors:** Luisa Jordao, Otilia V. Vieira

**Affiliations:** ^1^Department of Infectious Diseases, National Institute of Health, 1649-016 Lisbon, Portugal; ^2^Center for Neuroscience and Cell Biology, University of Coimbra, 3004-517 Coimbra, Portugal

## Abstract

Tuberculosis is an ancient infectious disease that remains a threat for public health around the world. Although the etiological agent as well as tuberculosis pathogenesis is well known, the molecular mechanisms underlying the host defense to the bacilli remain elusive. In this paper we focus on the innate immunity of this disease reviewing well-established and consensual mechanisms like *Mycobacterium tuberculosis* interference with phagosome maturation, less consensual mechanism like nitric oxide production, and new mechanisms, such as mycobacteria translocation to the cytosol, autophagy, and apoptosis/necrosis proposed mainly during the last decade.

## 1. Introduction

The history of tuberculosis (TB) mixtures with the history of humanity since TB is one of the oldest infectious diseases affecting mankind. Bone TB was identified in 4000 years old skeletons, from Europe and Middle East, as the cause of death, showing that this disease was already a widespread health problem back then. In recorded history, Hippocrates writes about patients with wasting away associated with chest pain and coughing, frequently with blood in sputum. These symptoms allowed Hippocrates to diagnose TB, which at that time was called “consumption”. The frequency of descriptions of patients with these symptoms indicated that the disease was already well entrenched in ancient times. 

During the 16th and 17th centuries, the explosion of the European population and the growth of large urban centres made this continent the epicentre of many TB epidemics. Although during the first half of the 19th century, the incidence of TB peaked, causing death to approximately one quarter of the European population, in the second half of this century, TB mortality decreased due to improving sanitation and housing. The 20th century brought a steadily drop of morbidity and mortality due to TB, in the developed world, due to better public health practices, massive vaccination with Calmette-Guérin bacillus (BCG) vaccine and the advent of antibiotics such as streptomycin. This downward trend ended in the mid-1980s, triggered by emergence of acquired immunodeficiency syndrome (AIDS) and an increase in homelessness and poverty in the developed world. This fact pointed to the important role played by the immune system in this disease and also to the importance of socioeconomical factors. More recently, we assisted to the identification of multidrug resistant (MDR) strains, defined as mycobacteria resistance to at least rifampicin and isoniazid (two first line anti-TB drugs) and extensively drug resistant (XDR) strains, defined as MDR mycobacteria with additional resistance to fluoroquinolones and at least one of the injectable second line antituberculosis drugs [1–3]. Notably, M/XDR-TB have been recognized by the World Health Organization (WHO) as the major challenge to be addressed in order to eradicate tuberculosis [4].

Currently, more than one-third of the world's population is infected with *Mycobacterium tuberculosis* (*M. tuberculosis*). According to the latest report released by the WHO, in 2009, there were 9.4 million incident cases, 14 million prevalent cases, 1.3 million deaths among HIV-negative people and 0.38 million deaths among HIV-positive people [3]. In addition to these already frightening numbers, people who are latently infected constitute the hidden reservoir of the disease from which new cases of active disease can emerge [3]. New effective drugs, against either replicating or latent bacilli, better vaccines, and new diagnostic methods are desperately needed to change and overcome this situation. Despite the big efforts made in order to develop new tools to fight this plague, no good candidates have been found. The first step towards this goal would be a better understanding of the host-pathogen relationship. In this paper we will focus on the progress that has been done on the study of *Mycobacterium*-host interactions and its importance for the understanding of tuberculosis pathogenesis as well as for the discovery of new therapeutic targets.

## 2. The Etiological Agent

The *Mycobacterium tuberculosis* complex includes strains of five species—*M. tuberculosis*, *M. canettii*, *M. africanum*, *M. microti,* and *M. bovis* and two subspecies—*M. caprae* and *M. pinnipedii* [5]. These mycobacteria are characterized by 99.9% similarity at nucleotide level and virtually identical 16S rRNA sequences [6–9] but differ widely in terms of host tropisms, phenotypes and pathogenicity [5, 10, 11]. 

The most notable member of the complex is *M. tuberculosis* the causative agent of human tuberculosis which has an exclusive tropism for this host. In contrast *M. bovis*, the etiologic agent of bovine tuberculosis, causes only 5%–10% of human tuberculosis cases with a pathobiology indistinguishable from the one caused by *M. tuberculosis* and a wider host spectrum. The impact of *M. bovis* in human health declined sharply after the advent of pasteurization but there are records of new cases among immunocompromised individuals as well as re-activation cases amongst elderly individuals [12]. The third member of the complex with an important, although geographically circumscribed, impact on human health is *M. africanum* which is responsible for half of the TB cases in West Africa [13–15].

## 3. The Pathogenesis of Tuberculosis

Tuberculosis is an airborne disease, since the infectious bacilli are inhaled as droplets from the atmosphere. In the lung, the bacteria are phagocytosed by the alveolar macrophages. The interaction of mycobacteria components with macrophage receptors, such as Toll-like receptors (TLRs) results in the production of chemokines and cytokines [16] that serve as infection signals. These signals result in migration of monocyte derived macrophages and dendritic cells from the blood stream to the site of infection in the lung. The dendritic cells that engulf bacteria then mature and migrate to the lymph nodes [17–19]. Once there, CD4 and CD8 T cells are primed against mycobacterial antigens. Primed T cells expand and migrate back to the focus of infection in the lungs, probably in response to mediators produced by infected cells. This phenomenon of cell migration towards the infection focus culminates in the formation of a granuloma, the hallmark of TB. The granuloma is formed by T cells, macrophages, B cells, dendritic cells, endothelial and epithelial cells, among others in a proportion that varies with its age. This granuloma prevents the spreading of bacilli resident within macrophages and generates an immune microenvironment which facilitates the interaction between cytokines secreted by macrophages and T cells. However, the granuloma also provides housing for *M. tuberculosis* during a long period of time. The latent bacilli can be later released if the cytokine balance is broken, triggering disease reactivation.

## 4. Mycobacteria Entry and the Triggering of Signalling Cascades into Host Cells

The interaction of *M. tuberculosis* with host cells is complex and far from being fully elucidated. The entry of *M. tuberculosis* into macrophages seems to occur via cholesterol-rich domains (rafts) of the plasma membrane [20], being mediated by receptor binding and phagocytosis. Despite the numerous *in vitro* studies that clearly identify different receptors involved in *M. tuberculosis* uptake, mainly by macrophages and dendritic cells [21, 22], the results obtained *in vivo* in receptor-deficient animals did not support the *in vitro* data [21, 23]. In this scenario, it is almost consensual that *in vivo* mycobacteria uptake is made by multiple receptors, such as C-type lectin receptors, complement receptors and scavenger receptors, rather than by a single receptor-mediated pathway, implying the activation multiple signalling cascades. 

The majority of the *in vitro* studies indicate that the bacilli favour interaction with complement and mannose receptors, which are benign, because they trigger minimal superoxide production. In contrast, mycobacteria uptake by Fc receptors, which play a minor role in the absence of specific antibodies [24], would trigger a vigorous host response and would establish a distinct intracellular trafficking pathway. This might explain why virulent mycobacteria avoid internalization by these receptors [21, 25]. However, the majority of experimental data suggest that the receptor type has little impact on intracellular survival of the bacteria [21, 22, 26]. 

The macrophage mannose receptors are expressed on mature macrophages and allow uptake of virulent *M. tuberculosis* H37Rv but not of avirulent H37Ra. The interaction between these receptors and mycobacteria seems to be mediated by the terminal residues present in mycobacteria lipoarabinomannam (LAM) [27, 28] that are also involved in CD14 interaction. Since the expression of these receptors is downregulated by gamma interferon (*γ*-IFN), their role in mycobacteria ingestion is restricted mainly to early stages of infection and to individuals with compromised cellular immunity [29]. In addition to mannose and complement receptors, other receptors such as surfactant protein A receptors [30], class A [26] and B scavenger receptors [23] and C-type lectin receptor (mincle) [31] are involved in mycobacteria uptake associated with a low proinflammatory response.

The Toll-like receptors (TLRs), which belong to the group of pattern recognition receptors (PRRs), are likely to be responsible for the immune recognition of pathogens in macrophages and thus for the pro-inflammatory cell signalling [32]. This class of receptors recognize pathogen associated-molecular patterns (PAMPs). In the case of mycobacteria, they recognize the main component of mycobacteria cell wall LAM [33] and trehalose 6,6′-dimycolate (TDM/cord factor) [34]. Interestingly, pathogenic mycobacteria avoid binding to this family of receptors by preventing a strong proinflammatory response at early stages of infection. 

A large number of TLRs were identified in mammals and two of them, TLR2 and TLR4, have been implicated in the activation of macrophages by mycobacteria involving MAP kinases (ERK 1/2, p38, and JNK), Janus kinase/signal transducer and activator of transcription (JAK/STAT) and NF-*κ*B pathways [34, 35]. The activation of these host-cell signalling cascades culminates with pro-inflammatory cytokines (such as IL-1, TNF-*α*, and interferons) and chemokine production. Pathogenic but not nonpathogenic mycobacteria have evolved mechanisms to suppress these signal transduction cascades and thereby attenuate the cytokine-induced immune response [34, 35]. 

Extracellular signal-regulated kinases (ERK 1/2) and p38 are members of MAP kinase family and became activated through the phosphorylation of tyrosine and threonine residues. Pathogenic mycobacteria such as *M. avium* and *M. tuberculosis* modulate MAP kinase activity. This leads to a decrease in pro-inflammatory response exemplified by a decrease in cytokine secretion such as TNF-*α* and their downstream effector and nitric oxide (NO). Since TNF-*α* receptors are true death receptors, the decrease in the production of this cytokine, induced by blockade of NF-*κ*B and MAP kinase activation, results in apoptosis inhibition. This outcome is extremely important, since apoptosis is believed to constitute an effective mechanism of intracellular mycobacteria killing [36] (discussed in more detail further in this paper).

## 5. Mycobacteria Persistence and Host Defence Mechanisms

Macrophages play a unique role in host response to mycobacterial infections. These cells represent both the primary effector cell for killing and the habitat in which mycobacteria reside. In order to survive pathogenic mycobacteria developed strategies to evade detection by the host immune system. Here, we discuss some of the most important strategies adopted by pathogenic mycobacteria to persist within macrophages.

### 5.1. NO and Reactive Nitrogen Radicals (RNI) Synthesis

The first microbicidal activity that any intracellular microbe will encounter within the macrophage is the oxidative burst [37]. This is a nonspecific immune mechanism triggered by microbes that results in the production of highly reactive chemical species known as reactive nitrogen intermediates (RNIs) and reactive oxygen intermediates (ROIs) [38]. Among ROIs, we found intermediate reaction products of O_2_ en route to water, namely, superoxide, hydrogen peroxide and hydroxyl radicals. In the case of RNIs the products correspond to molecular species in different oxidation states ranging from nitric oxide to nitrate. Among them is peroxynitrite, a powerful oxidant, originated from the reaction of an RNI (nitric oxide) with an ROI (superoxide) [38–40]. *Mycobacterium tuberculosis* has been shown to be highly resistant to ROIs, such as hydrogen peroxide [41] or hydroxyl radicals [42], and susceptible to RNIs such as nitric oxide (NO) [43], so we will focus in this paper on the latter.

NO generated in macrophages by the inducible nitric oxide synthase (iNOS: murine or by the human variant NOS2) and its derivatives are produced in response to bacterial infection. Pro-inflammatory cytokines (e.g., *γ*-IFN and TNF-*α*) and bacterial lipopolysaccharides (LPS) enhance NO synthesis [44–46]. The antimycobacterial effects of these intermediates were shown experimentally in macrophage cultures infected with *Mycobacterium* [47, 48]. Other studies in the murine model of infection involving iNOS inhibitors or mice with disruption in the gene *nos*2 highlighted the crucial role played by RNI in host defence against *Mycobacterium* infection [43, 49–52]. In contrast, the importance of NO and RNI in human defence against *M. tuberculosis* is a matter of controversy [53–56]. 

In conclusion, it seems that NO generated by iNOS or NOS2 is required for mycobacteria killing. However, it is unlikely that an effective killing would be achieved without delivery of bacteria to acidic compartments (late endosome/lysosomes) as suggested by the studies of different laboratories [51, 55]. Indeed, analysis of *γ*-IFN activated macrophages provided evidence that NO and RNI are insufficient to clear mycobacteria in the absence of acidification [55]. At low pH, NO bactericidal effects are boosted by conversion of nitrite to nitrous acid and its subsequent decomposition, culminating with the generation of NO [57, 58]. 

The *in vitro* tolerance of mycobacteria to RNI is strain, dose and time dependent, with pathogens being inherently more resistant than nonpathogens [58–61]. This suggests that pathogenic mycobacteria express genes that counteract the bactericidal or bacteriostatic effects of RNI. Different experimental approaches led to the identification of *no*xR1 and *nox*R3 which are able to confer RNI, and also ROI, resistance by a still unknown mechanism [62, 63]. Another gene involved in protection from oxidative stress is *ahp*C [64]. The product of *ahp*C, the alkyl hydroperoxide reductase subunit C (AhpC), can metabolise peroxynitrite anion into nitrite, thereby contributing to detoxifying this highly reactive species [65]. Peroxynitrite is a powerful oxidant produced by activated macrophages that can exert its toxic effects through protein modification [65, 66]. *In vitro* studies have shown that *M. tuberculosis* is resistant to this oxidant species but *M. smegmatis* and BCG are susceptible [67].

### 5.2. Phagosomal Maturation Arrest

Ingestion of invading microorganisms by phagocytosis is an essential component of the innate immune response. Phagocytosis is a multi-step process consisting of receptor-mediated recognition of particles which triggers signaling cascades responsible for extensive actin cytoskeletal rearrangement and membrane remodeling [68–70] culminating with particle engulfment. After internalization, the resulting phagosome undergoes maturation. This process involves sequential interactions with components of the endocytic pathway and culminates in fusion with lysosomes and formation of a phagolysosome [71]. The phagolysosome is an organelle with acidic pH, high content of hydrolases and defensins with the ability to generate toxic oxidative compounds, responsible for routine elimination of microorganisms [72, 73]. However, some microorganisms such as *M. tuberculosis* have developed the ability to arrest phagosomal maturation, thereby averting killing and causing infection [74].

**Figure 1:**
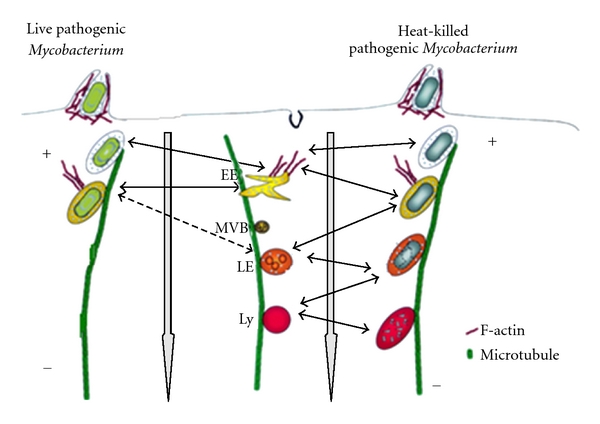
Maturation process of pathogen-containing phagosomes. Diagram outlining the differences between endosome progression to lysosome (center), maturation of a phagosome harboring a live pathogen, for example, *M. tuberculosis* H37rv (left) and a phagosome harboring a dead pathogen (right). The fusion processes of phagosomes containing live/dead pathogens with compartments of the endosomal pathway are indicated by bold arrows. The traced arrows indicate inhibition of the fusion events between the phagosome harboring the live pathogen and the endocytic compartment. The diagram also illustrates the importance of two components of the cell's cytoskeleton in phagosome maturation. Actin is recruited to the phagocytic cup and might nucleate on the phagosome membrane during the maturation process. The microtubules, to which endocytic vesicles and phagosomes are thought to bind during the maturation process, are also represented. EE: early endossome; MVB: multivesicular bodies; LE: late endosome; LY: lysosome. Diagram adapted from [71].

Phagosome maturation follows a defined biochemical program involving the sequential interaction with components of the endocytic pathway (Figure 1). The phagosome maturation involves both fusion and fission events that can be described by the kiss and run model of phagosome maturation [75]. A phagosome, which normally matures into the phagolysosome, fuses initially with early endosomes in an Rab5-dependent fashion and acquires the properties of this endocytic organelle. Thus, an early phagosome is characterized by the presence of Rab5 and its effectors such as the early endosome antigen 1 (EEA1), Class III PI3K, and its product phosphatidylinositol 3 phosphate [PI(3)P]. The transferrin receptor (TfR) is also present in the phagosomal membranes at this early stage. This organelle is also characterized by a relatively poor content of proteases and a mildly acidic pH of around 6. Some of these early markers are recycled from the phagosomal membrane back to the plasma membrane (such as TfR) as maturation proceeds [76, 77]. Thus, via fusion and fission events, phagosomes acquire new molecules and recycle others. Although the kinetics of maturation differ greatly and depend both on the particle phagocytosed and the cell, phagosomes begin to fuse with late endosomes and become refractory to early endosomes about 15–30 minutes after formation [71, 77, 78]. The loss of Rab5 and Rab7 acquisition enables subsequent fusion of the phagosome with older organelles, such as late endosomes and lysosomes [75, 79]. As a consequence of phagosome aging, they lose the early endocytic markers and become enriched in late endosome markers, which are best exemplified by Rab7, lysobisphosphatidic acid (LBPA), and the mannose-6-phosphate receptor cation independent [78, 80]. Nevertheless, the presence of these markers is also transient since the late phagosome evolves into a phagolysosome characterized by the presence of mature forms of lysosomal enzymes such as cathepsin D, lysosome associated membrane protein 1 (LAMP 1) and a luminal acidic pH ranging between 4 and 4.5 [71]. 

However, the use of more advanced quantitative techniques to evaluate the maturing phagosomal proteome revealed that the classical model of three consecutive fusions events with different endosomal systems (described above) is probably overly simplistic. Indeed, two quantitative proteomic studies [81, 82] have demonstrated that there are likely more distinct fusion events, presumably with subpopulations of the three main classes of endosomes suggesting that maturation is far more complex than a single Rab5 to Rab7 transition. Therefore, several other Rab proteins among other components of vesicular traffic such as SNARES, tethering factors and motor proteins must be integrated into this model to obtain a more complete map of the phagosomal maturation.

Intracellular pathogens have evolved highly specialised mechanisms to enter and survive within their hosts, resulting in devastating diseases. In order to do this, bacterial pathogens need to avoid host cell degradation and obtain nutrients and biosynthetic precursors, as well as evade detection by the host immune system [83–90]. The notorious success of *M. tuberculosis*, a facultative intracellular pathogen, rests upon the ability to arrest the biogenesis of the phagolysosome. The ability of this pathogen to enter host macrophages and persist in friendly phagosomes, which do not mature into phagolysosomes [89, 91–93], is crucial for tuberculosis infection, latency, disease activation, and spreading and suppression of immunological detection by the host [73, 74, 94, 95]. To create an intracellular niche that is favourable for replication, *Mycobacterium* inhibits the maturation of the phagosome by modifying its identity through the exploitation of host cell trafficking pathways. Indeed, following phagocytosis, the bacteria continue to reside within a membrane-bound vacuole of host origin. The seminal studies of D'Arcy Hart in the early 1970s described how the absence of fusion correlated with viability of the infecting bacteria [25, 91, 96]. The capacity of *M. tuberculosis* to regulate the fusogenicity of phagosomes is shared with other pathogenic mycobacteria such as *M. avium* and *M. bovis*. In 1986, Frehel and colleagues observed transient delivery of lysosomal tracers to phagosomes containing *M. avium* and suggested that these phagosomes had access to early endosomal compartment [97]. Moreover, in 1991 Crowle et al. [98] reported that phagosomes containing *M. avium* and *M. tuberculosis* were less acidic than neighbouring lysosomes. Furthermore, Sturgill-Koszycki et al. [99] reported that the pH of mycobacteria-containing phagosomes was around 6.2-6.3. Later on, it was shown that mycobacteria containing phagosomes are dynamic compartments with a paucity of V-ATPase complexes, which are responsible for the phagosomal acidification, and a profile of endosomal constituents consistent with the arrest of phagosome maturation at a point that retained fusion with early endosomes [100]. Markers of the recycling endosomal system, namely, the TfR, could also be shown to traffic through the mycobacteria-containing phagosome [100, 101]. Most researchers in this field are now working under the assumption that the phagosome containing pathogenic *Mycobacterium *is blocked at early stages of the maturation process [43, 73, 74, 92, 102–104]. It is thought that the maturation block occurs between the stages controlled by Rab5 and Rab7, being the latter excluded from the *M. tuberculosis* phagosome [80, 105, 106]. Of note, certain “lysosomal” markers, such as cathepsin D, could be detected in this compartment but a careful analysis revealed that it is an immature form of the enzyme [100, 107]. Thus, the strategies *M. tuberculosis* has developed to survive, and even replicate, inside the host is a subject of an intense investigation.

*Mycobacterium-*containing phagosome acquires Rab5 which in its active or GTP-bound state is able to recruit VPS34, a class III phosphatidylinositol 3-kinase responsible for PI(3)P synthesis. A model of how *M. tuberculosis* blocks phagosome maturation has emerged, based on altered VPS34 recruitment to mycobacterial phagosomes and altered PI(3)P patterns relative to the canonical model, latex bead phagosomes [78, 108]. PI(3)P is essential for phagosome maturation into a phagolysosome, and inhibition of PI(3)P production arrests phagosome maturation [78, 109]. Failure in PI(3)P synthesis by VPS34 can be attributed to the interference with the kinase activity by a Ca^2+^/calmodulin mediated process [110]. However, results published by Corvera and collaborators suggest that calmodulin does not affect VPS34 kinase activity but rather blocks EEA1 binding to PI(3)P [111]. More recently, Deretic's group [112] has shown that in addition to the known effects of *Mycobacterium* on suppressing Ca^2+^ fluxes [113–115], it also encodes a phosphatase that dephosphorylates PI(3)P and inhibits phagosome-late endosome fusion. These findings help to explain how live *M. tuberculosis* maintains the phagosome maturation block and avoids lysosomal compartments. Despite the finding that *M. tuberculosis* interferes with calmodulin and Ca^2+^-mediated signalling and the fact that it encodes a PI(3)P phosphatase, the controversy about the reasons behind the absence of PI(3)P in the phagosomal membrane during *M. tuberculosis* infection still persists. 

Although the majority of the studies on mycobacterial pathogenesis have been focused on Rab5 and Rab7, two GTPases that are known to play key roles in intracellular traffic, it is expected that other Rabs are also involved [116, 117]. Rabs regulate intracellular trafficking and maintain organelle identity by controlling incoming and outgoing cargo through budding, transport, tethering, docking, and fusion of vesicular intermediates, thus overseeing the vectorial transport of proteins and membranes between organelles [116]. If we take into account that in eukaryotic cells, organelle identity is determined, in part, by the composition of active Rab GTPases on the membranes, the retention or exclusion of Rab proteins from phagosomal membranes can also explain, at least in part, their escape from the degradative lysosomal pathways. Indeed, Rab10, Rab14, and Rab22 were also identified as contributors to the arrest of mycobacterial phagosomes by playing a role in the maintenance of *Mycobacterium *phagosome in its immature early endosomal-like stage [116, 118, 119]. Recently, it was also shown that Rab10 overexpression changed the properties of the *M. bovis *BCG-containing phagosomes. These authors reported that phagosomal membranes harboring BCG were acquired EEA-1, a marker excluded from the phagosomes in control cells (cells not transfected) [118].

### 5.3. Mycobacterium Translocation to the Cytosol

It is known that several intracellular pathogens such as *Listeria, Shigella*, *Ricketsia,* and *Trypanossoma cruzi *[83, 84] translocate to the cytosol in order to avoid degradation within the phagolysosome. This phenomenon was also described for *M. marinum*, which causes tuberculosis-like disease in their natural hosts, fish and frog [120] and “fish tank granuloma”, a granulomatous skin syndrome, among humans [121]. In infected cells, *M. marinum* is able to escape from the phagosome by a process not fully understood, in which pore formation in the *Mycobacterium *containing vacuole induced by the virulence factor early secreted antigenic target-6 (ESAT-6) might play an important role [122]. Once in the cytosol, *M. marinum* induces actin tail formation and initiates cell-to-cell spread. Although the mechanism of tail formation induced by *M. marinum* is largely unknown, there is evidence for the involvement of a nucleation promoter factor known as Wiskott-Aldrich syndrome protein (WASP), in a process dependent of the actin related proteins complex 2/3 (Arp 2/3) [123, 124]. More recently, another mechanism of cell-to-cell spread mediated by an actin-based structure called the ejectosome has been described for both *M. marinum* and *M. tuberculosis* in Amoeba (*Dictyostelium*). The specialized secretion system required for virulence (ESX-1), also responsible for secretion of ESAT-6, is involved in this process, which also encompasses coronin and Myosin II instead of Arp2/3 complex [125]. Clearly, more studies are needed to show whether the ejectosome and actin tail formation are concurrent or concerted strategies adopted by virulent mycobacteria to spread from one cell to another [126].

The escape of *M. tuberculosis* to the cytosol has been described by several groups [127–129] although none of them described the formation of actin tails. In all studies, the conclusions are based on the analysis of transmission electron microscopy data. In the two earlier studies, plastic embedding of the samples was performed [127, 128]. This technique did not allow the use of immunogold techniques for labeling with antibodies against the endocytic markers described in the previous section, such as LAMP-1 and EEA-1. However, despite the use of different types of macrophages both groups concluded that the nature of mycobacteria-containing compartment changed over time. Initially, mycobacteria were found in membrane enclosed compartments and after 1 [128] or 4 days [127] a significant number of *M. tuberculosis* H37rv was seen in the cytosol. In both studies a difference was also noticed in the behavior of mycobacteria with distinct virulence features. The virulent strain *M. tuberculosis* H37rv, translocates more efficiently to the cytosol than the attenuated strain *M. tuberculosis* H37ra or the vaccinal strain *M. bovis* BCG. However, the ability of *Mycobacterium tuberculosis* H37rv to translocate is lost when the bacilli are heat killed prior to internalization by macrophages. The explanation for these observations was provided years later by Van der Wel and colleagues [129]. In their work, advanced EM techniques, such as Tokuyasu cryo section and tomography were applied. These techniques allow immunogold staining and 3D reconstitution of individual mycobacteria, permitting more accurate conclusions. An additional strength of this work is the use of macrophages and dendritic cells derived from human monocytes, the natural host of *M. tuberculosis*. The authors showed that 2 days after infection *M. tuberculosis* progressively translocates from the phagolysosome to the cytosol and, once there, it is able to replicate faster than in membrane-enclosed compartments. This behaviour was not observed for BCG, in agreement with the previous studies. The explanation to this outcome is based in the genomic region of difference 1 (RD1), which is present in *M. tuberculosis* and *M. leprae* but deleted in BCG [130]. This genomic region characteristic of virulent mycobacteria encodes for virulence factors, such as the ESAT-6, which isalso secreted by *M. marinum* that translocates to the cytosol, and culture filtrate protein 10 (CFP-10).

### 5.4. Autophagy as an Immune Response to Mycobacterium Infection

Autophagy sometimes referred as “the art of self-eating” is a crucial process to cellular homeostasis and allows the cell to ingest and digest portions of its own cytosol, assuring an efficient “housekeeping” service [131]. This cellular process has three phases: initiation, elongation and closure, and maturation and is characterized by the emergence of a membranous organelle called the autophagosome (reviewed in [132]). This organelle captures cytosol components, such as defective organelles and large macromolecular aggregates, and intracellular pathogens such as *Toxoplasma gondii *[133], *Streptococcus* [134], *Shigella *[135], and *Mycobacterium* [136–140], delivering them for lysosomal degradation by different processes including ubiquitin mediated degradation [139] in autolysosomes. 

For *M. tuberculosis* it has been shown that autophagy induced pharmacologically or by starvation leads to mycobacteria delivery to lysosomes and subsequent killing [136]. Several key molecules for this process have been identified, including murine Irgm1 (LRG-47) guanosine triphosphate and its human orthologue IRGM, which is important for controlling *Mycobacterium* infections [137, 140]. In addition, PI(3)P, a key lipid in different cellular processes such as phagosome maturation, has been shown to be a central target for autophagy [141]. Although there is no doubt concerning the relevance of autophagy in innate and adaptive immunity in response to microbial infections, including by mycobacteria [142–144], more studies need to be performed in order to elucidate the molecular mechanisms and machinery involved in this process.

### 5.5. Host Cell Death and Mycobacterium Persistence: Apoptosis versus Necrosis

Recently, the induction of host macrophage necrosis, a type of cell death that favours *M. tuberculosis* survival within the host and is driven only by virulent *Mycobacterium* has been proposed as a novel virulence mechanism [145]. Cell necrosis is characterized by disruption of host surface membrane, facilitating escape of *M. tuberculosis* into the surrounding tissue for a new cycle of infection and dissemination from the lung to other tissues. In contrast, macrophage apoptosis, which is the alternative cell death modality, results in enhanced host defense by killing of intracellular *M. tuberculosis* and by boosting the adaptive immune response [146–149]. Indeed, macrophage apoptosis has been suggested as a novel defense mechanism against tuberculosis. Apoptosis of infected macrophages may act as an antimicrobial innate defense mechanism, and inhibition of apoptosis and induction of necrosis may serve as microbial virulence mechanisms. Apoptosis sequesters the pathogens within the cell, which facilitates efficient pathogen killing [150–152], promotes antigen presentation [153] and enhances microbicidal activity by macrophages and dendritic cells, that engulf apoptotic corpses [150]. 

The crucial role played by apoptosis in mycobacteria clearance was shown by the discovery of Irp1 gene [154]. Mice lacking this gene are extremely susceptible to *M. tuberculosis* and their infected macrophages undergo necrosis. In contrast, expression of Irp1 limits *M. tuberculosis* replication and leads infected macrophages toward apoptosis. The antimycobacterial mechanisms triggered by apoptosis are complex and heterogeneous. In early studies, it was suggested that the integrity of the genetic material of the bacilli could be compromised [155] and the control of acidification and fusion of *Mycobacterium*-containing phagosomes were subverted during apoptosis, depriving the pathogen from its intracellular niche [156]. More recently, it was proposed that enhanced killing of mycobacteria in apoptotic macrophages could be driven by stabilization of mitochondrial permeability transition [150] or by an ATP/P2X_7_ purinergic receptor apoptotic-mediated mechanism not yet fully elucidated. This process probably involves enhancement of *Mycobacterium *containing phagosome fusion with lysosomes [157], in accordance with previously reported observations [156, 158]. Another possible mechanism for P2X_7_ mediated mycobacteria killing is the crosstalk between apoptosis and autophagy [159]. 

Several mechanisms have been proposed to explain apoptosis inhibition by *M. tuberculosis*. The interference with TNF-*α*, a cytokine that plays a key role in mycobacteria pathogenesis [95], was among the first mechanisms proposed [160, 161]. It was suggested that *M. tuberculosis* evades apoptosis of host macrophages by inducing the release of soluble TNFR2 which complexes with TNF- *α* and decreases its activity in an IL-10-dependent manner [162]. Another hypothesis explored was the ability of pathogenic *M. tuberculosis* to alter the permeability of macrophage mitochondrial membrane in such a way that favours necrosis instead of apoptosis [145]. More recently, it was reported that the mechanisms determining whether infected macrophages undergo apoptosis or necrosis relay on two distinct lipid-mediators of host signaling, PGE_2_ and LXA_4_. These lipid mediators have a common precursor, arachidonic acid (AA). AA is released from phospholipids present in the cytoplasmic membrane by the cytosolic enzyme phospholipase A_2_ [163] and its degradation by either 5-lipoxigenase or cycloxygenase 2 generates LXA_4_ or PGE_2_, respectively. Although lipids such as eicosanoids play an important role in disease (for review see [164]) and particularly in tuberculosis [165], the mechanisms triggered by mycobacteria to control lipid metabolism are far from being fully elucidated. Virulent *M. tuberculosis* induces the production of LXA_4_, which suppresses PGE_2_ synthesis and leads to macrophage necrosis. In contrast, avirulent *Mycobacterium* induces only small amounts of LXA_4_ production in infected macrophages. Instead, these infected cells produce PGE_2_, which results in cellular apoptosis rather than necrosis. Furthermore, *Alox*5^−/−^ mice, which are unable to synthesize LXA_4_, have a greater ability to control virulent *M. tuberculosis* infection compared to wild-type mice [146]. In addition, in trying to elucidate a key downstream event modulated by these distinct modes of lipid-mediated signaling, plasma membrane (PM) disruptions of infected macrophages induced by *M. tuberculosis* infection were reported [148, 166]. Importantly, whereas infection by avirulent forms of *M. tuberculosis* allows the host to repair these PM defects, virulent infection blocks this host repair process. As a result of this block, infected macrophages undergo necrosis rather than apoptosis. Taking clues from elucidated mechanisms of PM repair that occur in other settings, such as traumatic disruption of the PM, it was further defined that membrane transport from the Golgi and lysosome contributes to host PM repair that occurs during avirulent infection [144]. Moreover, these transport pathways become blocked during virulent infection. Thus, membrane repair seems to be a critical mechanism that results in impermeability of the apoptotic macrophage leading to containment of *M. tuberculosis *and its products within the phagosome. In conclusion, the ability of *M. tuberculosis* to precisely modulate the outcome of a programmed cell death process probably represents one of the important immune-evasion strategies that make this organism such a formidable challenge to global health.

## 6. Final Remarks

Despite the intensive work on the mycobacteria field and the use of cutting-edge technology, such as EM techniques and genome-wide screenings for mycobacteria and host, many questions remain unanswered. This can be explained by the *Mycobacterium* complexity and its ability to adapt to the host environment. Indeed, *M. tuberculosis* has a variety of important immune-evasion strategies representing one of most challenging pathogen to mankind. Among these strategies is the subversion of host membrane machinery that is important for the uptake, survival, and replication of this pathogen. In the future, the identification of all the host machinery involved in phagocytosis and phagosomal maturation will certainly help in understanding how *Mycobacterium* manipulates host membrane transport pathways, providing mechanistic insights into how infection occurs and revealing new information on biochemical processes involved in the functioning of the host cells.
